# Supplementary material for: Healthy Nutrition and Physical Activity in Childcare: Views from Childcare Managers, Childcare Workers and Parents on Influential Factors
Source: Int J Environ Res Public Health. 2018 Dec 19;15(12):2909. doi: 10.3390/ijerph15122909 (PMC6313341; doi:10.3390/ijerph15122909)
Supplement: Supplementary file 1 [file ijerph-15-02909-s001.pdf]

Supplementary Materials

Table S1. Concepts and quotes illustrating the concepts identified in the interviews.

**Concepts**	**Quotations to illustrate the concepts identified**
Intrapersonal factors - attitude	
Importance of healthy living	'Because it's healthier for your body, that you have more energy and I just think it is good to start with that at a young age.' (CW4.1)<br>'As an extra they can have something [juice], but rather only water … I think that's important for health reasons.' (P20)<br>'We often come and watch and they love to play outside … they are so actively playing; it is nice to see.' (P33)<br>'What I particularly find important is that they go outside in nice weather, that a child can be a child.' (P18)<br>'So, we don't have the rule that everyone has to be seated at the table. They are only toddlers, right? It should not be that this is already school-like, that children grab a puzzle, sit at the table, clean up and go to the next one.' (CW8.2)
Balance	'You have to be able to sometimes eat something sweet and to snack a bit, you have to be honest in that.' (CW3.1)<br>'Well, my philosophy is that if you do not give it to children when they are young, do not let them experience it, they will not know how to control themselves.' (CM1)<br>'I do not think it is a problem. You see, he needs to get nice things, and he needs to get healthy things, there has to be a balance in it.' (P27)
Intrapersonal factors – Social norm	
	'Not all parents are too happy with that. That is something striking: if it is cold, you do not go outside.' (CW2.1)<br>'What I say with the candy, but we have been doing it for ages. And we already say it [that healthy treats are preferred] but it does not happen, so we actually have given up on it.' (CW6.2)<br>'You can see that an increasing number of people are being more conscious about nutrition and physical activity.' (CM6)<br>'You indeed notice that more and more parents ask questions or say "I do not want them to participate with birthday treats". Parents are very, very occupied with it and, therefore, we are also very occupied with it.' (CM5)
Intrapersonal factors – Perceived behavioural control	
	'Besides the time constraints, you also have to comply with all sorts of guidelines. The Community Health Service says, "Before you eat fruit, the children have to wash their hands for at least 30 seconds."' (CW4.2)<br>'You can only do that if you have a smaller group, six, seven children, max eight … Most of the time the groups are totally full.' (CW8.1)<br>'I do think it is very important to get advice in it. I am not clumsy, and I know a lot about what they do at schools. But still I find it difficult.' (P28)<br>'But we know how to make our way in that.' (P17)<br>'No, not at all. She [child] is used to drinking water, so she likes it.' (P16)
Environmental factors – Physical environment	
Availability	'If you see that big play area, you would think that it would fit some more [play materials], on the other hand you could also just organize an activity. So it also provides opportunities.' (P23)

	'We have specified timeslots and in the afternoon it is in consultation, mostly we can use it [indoor physical education room] … we have to adjust, and it does not always suit well with snack time for example.' (CW3.2)<br>'What we really encounter is challenge for the children. We can do a lot of games, but they also like to do something for themselves, and then you have five crappy bikes and a sandpit in which the sand does not get replaced and two balls. So, we do come short in relation to play materials.' (CW6.1)<br>'They have bikes and a sandpit what I saw. Some small play materials, and inside it is a paradise anyway. So they learn with puzzles, drawings, painting, all sorts of things.' (P13)
Variety	'Well, they take a piece of fruit with them, but you see that they are very selective in what they take. The most things that they eat are bananas and apples, with kiwifruit it is already more difficult, tomatoes, oranges, tangerines. Some find it nice, and others do not, so you notice that there is not much variety they can choose from.' (CW4.1)<br>'They get the usual pieces, pear, apple, banana and oranges and tangerines, so this is quite varied already.' (CW7.2)<br>'I think that there is great variety in what they get offered from what others bring.' (P22)
Space	'But that is also just not possible here, because if they start running, you already have to warn them "be careful with the tables."' (CW1.1)<br>'We adjusted the outside play areas, so that they [children] can be outside independently and play outside. Everything is fenced and safe in order to let them be outside as much as possible.' (CM3)<br>'I think there is enough physical activity, but I do not think there is enough space. I find it quite small outside, certainly for a group of sixteen children. Yes, I find it quite tight.' (P13)
Environmental factors – Sociocultural environment	
Nutrition and physical activity related practices	'What we do occasionally, when we have a lot of tangerines, we let the children peel the tangerines.' (CW4.1)<br>'We also give a good example. If fruit is eaten, then we also eat a piece of fruit.'(CW8.2)<br>'Most of the activities that we do [during circle time] is not only sitting. This morning we did something with colours and placed all colourful papers throughout the classroom … and then the children have to walk around and search for all the yellow cards.' (CW9.2)<br>'If they do not like tangerine, then I also take a piece of tangerine, and then we do it together. And sometimes I see that the child has it in their mouth, although he might not eat it. But he did chew on it and, well, then he did taste it already.' (CW9.2)
Influences on practices	'I see and I hear that we also have some childcare workers, if there is a team with some that just do not want to go outside, then it also does not happen.' (CM4)<br>'Indeed, they [childcare workers] like to go outside to let the children play, but with more structured activities, you see that particularly the older childcare workers find that more difficult, for example, to come up with a game.' (CM5)<br>'What we do see with playing outside is that sometimes a child is not used to it … that they kind of have to learn here to play outside.' (CW2.2)

	'For example, a child enters with a sandwich in the hand, because he does not want to eat at home. Yes, what is not wanting to eat, he is asleep longer right? Well, that is a choice made, so he gets a sandwich shoved in his hand, and he is supposed to eat that here.' (CW4.1)<br>'Some parents ask, "Do they eat at the table?" Yes, they all sit at the table, for some parents that is unimaginable.' (CW8.2)
Environmental factors – Economic environment	
Childcare budget	'We just do what we think is important, look if bikes are worn out, then we make sure that there will be new bikes.' (CM1)<br>'We just buy something if we want to have it. If we think it is a nice offer and we can really use it, we buy it. We do not really think in percentages and budgets.' (CM2)<br>'There is no money for a lot of things, that is the standard reply.' (CW8.2)<br>'But that is just because of the money and not because of safety.' (CW9.2)<br>'It depends, if it is not too costly, otherwise we have to request it… but if it is just something like that balance beam, I just get it myself.' (CW10.1)<br>'I think parents pay enough tuition, so we could provide fruit. If I look at my son who is at day care, he gets his food all day, and I think this should also be the case for us, honestly.' (CW2.1)
Financial situation of parents	'You cannot force parents to bring a pineapple if that puts someone to great expense.' (CW6.1)<br>'Like strawberries or mangos, that is more expensive fruit, and I cannot expect that from parents, and I will not ask for it.' (CW10.1)
Environmental factors – Political environment	
Childcare policies	'But from our organization there is actually only the advice to always give fruit and nothing about drinks.' (CW8.1)<br>'I talked about it with my manager, and I would appreciate having a policy from the organisation that birthday treats are no longer necessary instead of one half does it and the other half does not.' (CW3.2)<br>'Although it is written in the rules and although we say to parents try to think of something else, we do not send anyone home who still brings sweets.' (CW4.2)<br>'It is something that goes automatically if you find it important. Yes, we do not really have that in a policy, it is just we know … in the regular schedule there are at least two moments of physical activity and play. Often it is much more.' (CM1)<br>'We looked consciously at our nutrition policy, what is and is not bought at the facilities and ensuring the same things are done at the facilities, that birthdays are celebrated in the same way. That parents do not bring the treats, but that we do that ourselves.' (CM2)
Rules and regulations of the Community Health Service	'Because it has to be durable, it has to comply with all regulations. It cannot cause splinters, it has to be fixed, be closed and around it, it has to be safe.' (CW5.2)<br>'It [safety] has to be covered from all sides. That you start to notice, also for myself that you are sometimes afraid to do something like what if one of them slips or…' (CW8.1)
Cross-setting influence between childcare – home setting	
	'You can pick out the children who did not have breakfast immediately… that is really stuffing, and I think yeah this is probably the first thing you get today.' (CW2.1)

	'Do you remember that we, when there was snow, we actually wanted to go outside with the children. But many do not wear shoes that are suitable or wear a winter coat … we really have to promote to make sure children wear a coat.' (CW8.1)<br>'We do make exceptions for parents with certain wishes… there is a group of children of which the parents say, "They cannot have milk, they really cannot have sweets, they do not participate in birthday treats, they drink just water," and then we go with that… yes, those children are an exception to the rest of the group.' (CM5)<br>'It has grown in the past years, also with parents and I do it too myself at home. Then I hear myself talking, because we are now so aware of it, "Be careful, watch out, that makes you dirty."' (CM6)
Moderators	
Demographics	'We have some families that are in debt restructuring, and they get a limited budget. You could eat healthily, but it will definitely have an influence.' (CW4.2)<br>'Yeah, that depends also [healthy treats or not], not to be judgemental, but it depends on the background.' (P18)<br>'What we do come across is that foreign parents, who do not speak Dutch well, so you cannot communicate well, that they have their own customs.' (CW5.2)<br>'There are children who eat more couscous than potatoes and, well, the couscous may be equally healthy, but it influences what you eat.' (CW4.2)<br>'If you have a more multi-cultural day care, then things are different with nutrition.' (CM1)<br>'It is safer to have babies and toddlers alone outside, than when there are also three- and four-year-olds with balls and all materials. So, that was a barrier, and we arranged our spaces around it.' (CM3)
Child characteristics	'That many children have problems eating, do not like everything and that because of that there is little variety in what is offered, that is something you clearly notice.' (CW1.1)<br>'Children do have a preference for certain things. We have those periods that we leave the skin on the apples, but you notice that they do not like that.' (CW6.2)<br>'We have bikes and a sandpit and then you see that children who are a little bigger or do not like being active that much, that they sit in the sandpit and you will say, "Come on, on these bikes", but it does not happen.' (CW5.1)<br>'We actually thought that it would be one big playground, but the fence was kept in place. And well, you can divide it between children who are quicker and children who want to play more carefully.' (CW7.1)<br>'Well, they say not to bring strawberries or kiwi fruit because of allergies.' (P28)
Habit strength	'I would not know what should be done differently, we have been doing it for years.' (CW5.1)<br>'Something that is also nice is to just give something, a little toy or something. Children always like that too… but it [treating with sweets] is just what they're used to.' (P11)<br>'That is the whole lifestyle at home, they are used to it like that from growing up. That is something that you should change.' (CW2.2)
Awareness	'Sometimes I even think was that really that bad? That I only now start to realise how much [sugar] it actually contains.' (CW3.2)

	'You can be proud that you are allowed to help them grow and being aware of physical activity and healthy nutrition is part of that.' (CM1)
